# Towards understanding π-stacking interactions between non-aromatic rings

**DOI:** 10.1107/S2052252519000186

**Published:** 2019-02-02

**Authors:** Krešimir Molčanov, Biserka Kojić-Prodić

**Affiliations:** a Department of Physical Chemistry, Rudjer Bošković Institute, Bijenička 54, Zagreb 10000, Croatia

**Keywords:** π-stacking interactions, non-aromatic rings, multicentric bonding, charge

## Abstract

The first systematic study of π interactions between non-aromatic rings, based on the authors’ own results from an experimental X-ray charge-density analysis assisted by quantum chemical calculations, is presented. The landmark (non-aromatic) examples include quinoid rings, planar radicals and metal-chelate rings. The results can be summarized as: (i) non-aromatic planar polyenic rings can be stacked, (ii) interactions are more pronounced between systems or rings with little or no π-electron delocalization (*e.g.* quinones) than those involving delocalized systems (*e.g.* aromatics), and (iii) the main component of the interaction is electrostatic/multipolar between closed-shell rings, whereas (iv) interactions between radicals involve a significant covalent contribution (multicentric bonding). Thus, stacking covers a wide range of interactions and energies, ranging from weak dispersion to unlocalized two-electron multicentric covalent bonding (‘pancake bonding’), allowing a face-to-face stacking arrangement in some chemical species (quinone anions). The predominant interaction in a particular stacked system modulates the physical properties and defines a strategy for crystal engineering of functional materials.

## Abstract 2

The first systematic study is presented of π interactions between non-aromatic rings, based on the authors’ own results from an experimental X-ray charge-density analysis assisted by quantum chemical calculations. The study includes quinoid rings, planar organic radicals and metal chelates. The stacking observed covers a wide range of interactions and energies, ranging from weak dispersion to unlocalized two-electron multicentric covalent bonding (‘pancake bonding’).

## 1. Introduction   

π-Stacking of aromatic rings is a well known type of intermolecular interaction which has been studied extensively over the last few decades (Wheeler & Bloom, 2014 ▸, and references therein) and applied in supramolecular chemistry (Steed & Atwood, 2009 ▸) and crystal engineering (Desiraju *et al.*, 2011 ▸; Tiekink & Zukerman-Schpector, 2012 ▸). It plays a significant role in the crystal packing of aromatic compounds (Steed & Atwood, 2009 ▸; Groom *et al.*, 2016 ▸) and the properties of functional materials (Bredas *et al.*, 2011 ▸; Carini *et al.*, 2017 ▸). These interactions are of great significance in molecular recognition in biological systems (Salonen *et al.*, 2011 ▸; Riley & Hobza, 2013 ▸; Madhusudan Makwana & Mahalakshmi, 2015 ▸; Neel *et al.*, 2017 ▸). They stabilize the DNA helix (Mak, 2016 ▸) and they are involved in interactions between drugs and proteins (Bissantz *et al.*, 2010 ▸; Wilson *et al.*, 2014 ▸). Stacking interactions also help to bind hydro­phobic ligands onto the active sites of enzymes (Stornaiuolo *et al.*, 2013 ▸). Recently, and unexpectedly, evidence has come to light of one further role of aromatic stacking: it is a key step in nucleation kinetics during crystallization experiments, overpowering hydrogen bonding (Cruz-Cabeza *et al.*, 2017 ▸).

π-Stacking interactions can be modulated by chemical modifications, crystal engineering and external stimuli, and therefore they are a subject of extensive research in materials science, particularly in carbon nanostructures involving fullerenes, carbon nanotubes and graphene (Pérez & Martín, 2015 ▸).

However, a consensus for an appropriate term has yet to be reached and currently quite a variety of names are used by different authors: π–π interaction, π interaction, π stacking, stacking interaction, aromatic interaction, σ–π interaction, aromatic–aromatic interaction, aryl interaction *etc*. Some authors are opposed to the above terms, advocating for a more specific and detailed description of the aromatic system and interaction forces (Grimme, 2008 ▸; Martinez & Iverson, 2012 ▸; Wheeler & Bloom, 2014 ▸).

**Figure 1:**
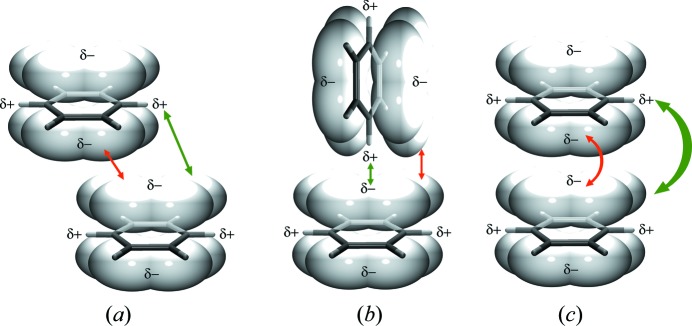
The quadrupolar model of stacking of aromatic rings proposed by Hunter & Sanders (1990 ▸). Energetically favourable arrangements of the rings are (*a*) parallel and offset or (*b*) T-shaped. The face-to-face arrangement shown in panel (*c*) is energetically unfavourable due to strong repulsion between the π-electron clouds. The typical geometry for type (*a*) is a centroid-to-centroid distance >3.8 Å, an interplanar distance >3.5 Å and an offset of *ca* 1.7 Å.

According to the model of Hunter & Sanders (1990 ▸), π interaction is essentially an attractive interaction of electrical quadrupoles, which overpowers the repulsion of π-electron clouds (Fig. 1 ▸). In parallel, offset and T-shaped arrangements (Fig. 1 ▸), the total interaction is slightly positive, since σ–π attraction is stronger than π–π repulsion. The π-polar model (Hunter *et al.*, 2001 ▸) of aromatic interactions has been refined over time, taking into consideration direct substituent interactions and solvation/desolvation effects (Hunter & Sanders, 1990 ▸; Janiak, 2000 ▸; Hunter *et al.*, 2001 ▸; Salonen *et al.*, 2011 ▸; Martinez & Iverson, 2012 ▸; Wheeler & Bloom, 2014 ▸; Carini *et al.*, 2017 ▸). Face-to-face stacking is possible between electron-rich aromatics and electron-depleted ones such as hexa­fluoro­benzene (also an example of double aromaticity arising from π-orbital and σ-orbital interactions; Furukawa *et al.*, 2018 ▸) and is often referred to as an aromatic donor–acceptor interaction; some level of π-orbital mixing occurs and the donor–acceptor term better describes a situation in which relatively electron-deficient and electron-rich aromatic mol­ecules stack in an alternating fashion (Hunter & Sanders, 1990 ▸; Martinez & Iverson, 2012 ▸). These cases are, however, rare.

In addition to classical π stacking of two aromatic systems (including heteroaromatics), there are numerous examples of hetero π stacking involving a non-aromatic stacking partner (Neel *et al.*, 2017 ▸): (i) *X*H pointing towards the centroid of the aromatic ring (*X* = B, C, N, O, halogen) (Hunter & Sanders, 1990 ▸; Bloom *et al.*, 2012 ▸; Neel *et al.*, 2017 ▸), (ii) ions (Quiñonero *et al.*, 2006 ▸; Neel *et al.*, 2017 ▸) and (iii) a lone pair (Carini *et al.*, 2017 ▸; Neel *et al.*, 2017 ▸; Newberry & Raines, 2017 ▸). One recent result based on experimental and theoretical evidence has revealed dimer stacking through a π-pyrrole⋯π-(N_2_) interaction that energetically overpowers hydrogen bonding (Ramanathan *et al.*, 2017 ▸). An even more complex interaction of hetero π stacking involves a lone pair (lp) as a partner (lp-π, known as *n*→π*), representing a nucleophile lone-pair donation to an empty π* orbital (Quiñonero *et al.*, 2006 ▸; Neel *et al.*, 2017 ▸). A ubiquitous example is the carbonyl group acting as an lp partner in stacking occurring in numerous chemical reactions in chemistry and biology; interaction between the lone pair of the carbonyl group and a π system is an important factor in the stabilization of protein conformations (Quiñonero *et al.*, 2006 ▸; Neel *et al.*, 2017 ▸).

However, there are many types of chemical system which are neither aromatic nor anti-aromatic (Nozawa *et al.*, 2016 ▸), which do not obey Hückel’s rule of (4*n*+2) π or (4*n*) π electrons, respectively. They do not meet any of the criteria for these two categories but they can stack, as demonstrated by our examples of molecules belonging to different chemical classes: quinones (including charged ones), a variety of planar organic radicals, including semi­quinones and tetra­cyano­ethyl­ene, and metal-chelate rings. Our findings are in agreement with the observation that more favourable stacking interactions can be achieved by exploiting the interactions of non-aromatic polyenes rather than aromatic systems, an idea put forward by Bloom & Wheeler (2011 ▸) and developed further by Wheeler (2013 ▸).

## 2. Discussion   

In the present paper we emphasize that the presence of a stacking motif does not depend on the presence of an aromatic system. Different chemical species comprising π systems that are inclined to stacking arrangements can include a plethora of intermolecular interactions. The examples studied include quinoid rings, semi­quinone anion radicals and their combinations, and metal-chelate rings (Scheme 1); the study is mostly based on our experimental determinations of X-ray charge density, including atoms-in-molecules (AIM) analysis, supported by quantum chemistry models. In the stacks of closed-shell molecules with little or no electron delocalization (such as quinones), the interactions are much stronger, whereas for fully delocalized π systems (*i.e.* aromatics) they are weak.
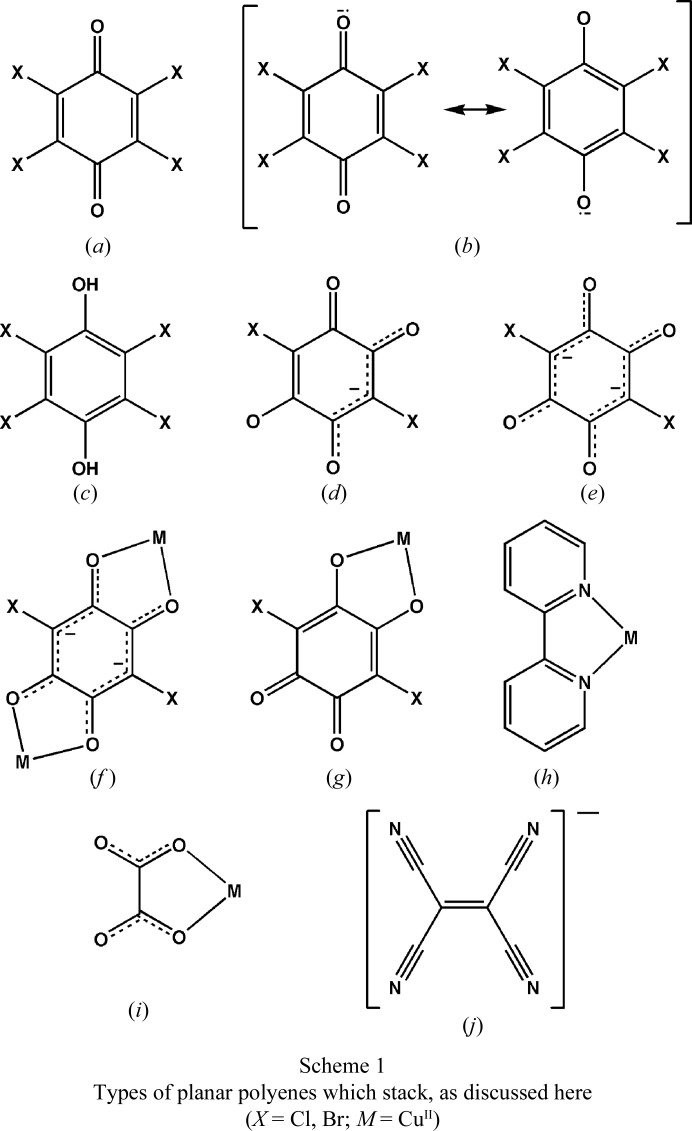


For example, stacking of organic radicals and charge-transfer compounds has been used for the design of magnetic (Itkis *et al.*, 2002 ▸; Hicks, 2011 ▸; Sanvito, 2011 ▸) and conductive molecular materials (Podzorov, 2010 ▸; Lekin *et al.*, 2010 ▸; Yu *et al.*, 2011 ▸, 2012 ▸; Nakano, 2014 ▸; Chen *et al.*, 2016 ▸) for more than a decade. Recently, it has been documented that unusually short and strong interactions between planar radicals have a partial covalent character (Huang & Kertesz, 2007 ▸; Huang *et al.*, 2008 ▸; Novoa *et al.*, 2009 ▸; Tian & Kertesz, 2011 ▸; Cui *et al.*, 2014*a* ▸,*b* ▸; Preuss, 2014 ▸) and this type of interaction has been termed ‘pancake bonding’. In our previous analysis of the stacking interactions of the semi­quinone radical, using X-ray charge-density analysis, ‘pancake bonding’ was described in detail (Molčanov *et al.*, 2019 ▸).

### 2.1. Stacking of quinoid rings: interactions of electrical multipoles   

**Figure 2:**
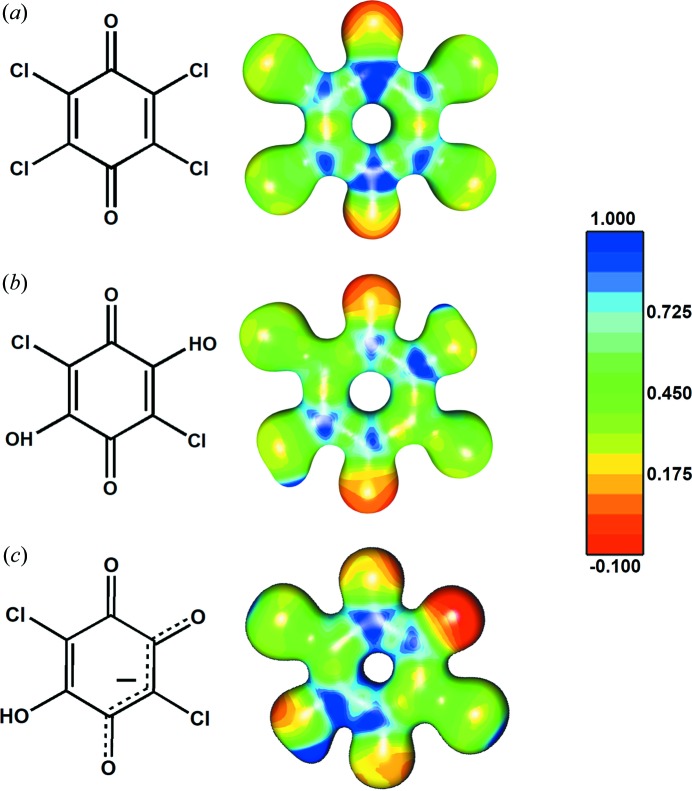
Electrostatic potentials plotted onto electron-density isosurfaces of 0.5 e Å^−3^ for (*a*) tetrachloroquinone (Molčanov *et al.*, 2019 ▸), (*b*) neutral chloranilic acid (Vuković *et al.*, 2019 ▸) and (*c*) the hydrogen chloranilate monoanion (Molčanov *et al.*, 2015 ▸). The electrostatic potentials range from −0.1 e Å^−1^ (red) to 1.0 e Å^−1^ (dark blue). Alternating electron-rich and electron-depleted areas can be observed.

Quinoid rings are not aromatic: the harmonic oscillator model of aromaticity (HOMA) indices of benzo­quinone (BQ) and tetra­chloro­quinone (Cl_4_Q) are −0.61 and −0.95, respectively (Molčanov *et al.*, 2011 ▸ *a*). Unlike aromatics, they have distinguishable single and double C—C bonds. Therefore, the electrostatic potential in the rings is not uniform, but the molecules contain alternating electron-rich and electron-depleted areas (Fig. 2 ▸), so the quadrupolar approximation is no longer valid. However, the rings’ charge density can be described using the multipolar expansion (*i.e.* by treating their atoms as a series of multipoles).

**Figure 3:**
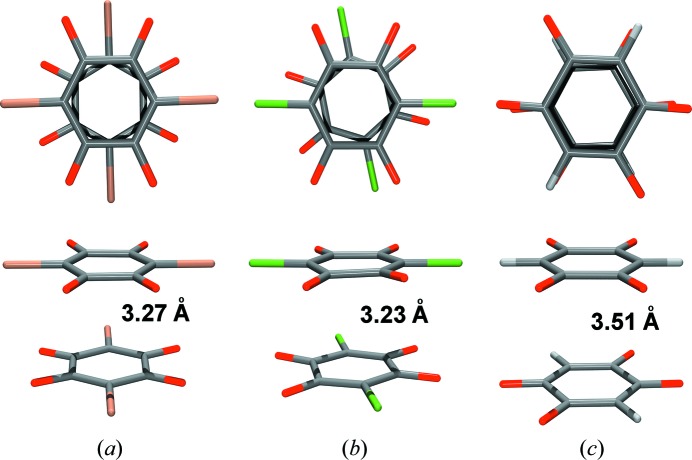
Pairs of contiguous quinoid rings in face-to-face stacks. (*a*) Hydrogen bromanilate monoanions (Molčanov & Kojić-Prodić, 2012 ▸) in a staggered arrangement. (*b*) Hydrogen chloranilate monoanions (Molčanov *et al.*, 2009*b* ▸) in a partially staggered arrangement. (*c*) DHQ^2−^ dianions in an eclipsed arrangement (Molčanov *et al.*, 2013*b* ▸). Figure adapted from Molčanov *et al.* (2013*b* ▸).

**Figure 4:**
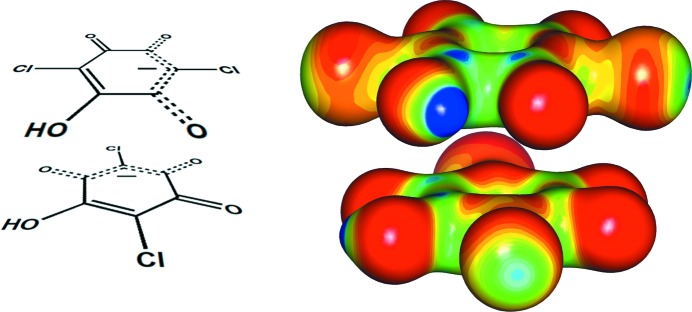
The electrostatic potential in a pair of contiguous face-to-face stacked hydrogen chloranilate anions in the crystal packing of KHCA·2H_2_O (Molčanov *et al.*, 2009*b* ▸) mapped onto an isosurface of 0.35 e Å^−3^, showing the interactions between the electron-rich (red and orange) and electron-poor (blue, green) regions (red: −0.35, blue: 0.35 e Å^−1^). Figure reproduced with permission from Molčanov *et al.* (2015 ▸), copyright (2015) Royal Society of Chemistry.

Since stacking interactions are, to a large extent, determined by charge density, we might expect that quinones would not stack according to the quadrupolar Hunter–Sanders model. 2,5-Di­hydroxy­quinones (DHQs) and their anions favour face-to-face stacking with a short (*ca* 3.3 Å) interplanar separation (Molčanov *et al.*, 2009*b* ▸, 2011*b* ▸, 2013*b* ▸; Molčanov & Kojić-Prodić, 2012 ▸) (Fig. 3 ▸). Crystal-packing analysis based on distance criteria can classify this interaction as a strong one. However, to gain insight into the character of interactions involving stacked pairs, additional more quantitative evidence is needed. Therefore, we studied face-to-face stacking in the model compound potassium hydrogen chloranilate dihydrate (KHCA·2H_2_O) (Molčanov *et al.*, 2011 ▸ *a*) by a combination of X-ray charge-density analysis and quantum chemical computation (Molčanov *et al.*, 2015 ▸). The electrostatic potential in a pair of rings (Fig. 4 ▸) shows an excellent fit of the electron-rich and electron-depleted areas of two contiguous rings, maximizing the electrostatic attraction while minimizing repulsion. However, the low electron density of only 0.05 e Å^−3^ indicates a closed-shell interaction. Its energy, estimated by second-order Møller–Plesset theory (MP2) calculations of isolated clusters and simulation of the crystal lattice by periodic density functional theory (DFT), is about −10 kcal mol^−1^ (Molčanov *et al.*, 2015 ▸; 1 kcal mol^−1^ = 4.184 kJ mol^−1^). While this is only a fraction of the electrostatic repulsion of negative charges, the stacks are held together by Madelung energy (the negative charges are compensated by nearby cations, and the attractions prevail).

However, the chloranilate and bromanilate dianions (CA^2−^ and BA^2−^, respectively) do not form face-to-face stacks, but stack in an offset fashion, with geometries similar to aromatic stacks (Molčanov *et al.*, 2009*a* ▸, 2011*a* ▸; Molčanov & Kojić-Prodić, 2012 ▸). This is partly due to the increased repulsion of double negative charges and partly because more electron delocalization does not allow a good match between the electron-rich and electron-poor parts of the contiguous rings.

Interestingly, unsubstituted DHQ^2−^ dianions in their simple alkali salts do stack face-to-face (Molčanov *et al.*, 2013*b* ▸), albeit with larger interplanar separations (exceeding 3.5 Å) due to repulsion of the double negative charges. The ideal face-to-face fit is here facilitated by close contacts of electron-rich C=O π bonds and electron-depleted C—H σ bonds [Fig. 3 ▸(*c*)]. Quantum chemical calculations indicate that the energy of interaction is at least −6 kcal mol^−1^ (Molčanov *et al.*, 2013*b* ▸). It is interesting to note that the steric effect of the substituents determines the orientation of the rings, so hydrogen bromanilate (HBA^−^) anions form perfectly staggered stacks (the anions are rotated by 30°) while DHQ^2−^ dianions are perfectly eclipsed (rotated by 0°) (Fig. 3 ▸).

### 2.2. Stacked radicals involve unlocalized covalent inter­actions   

**Figure 5:**
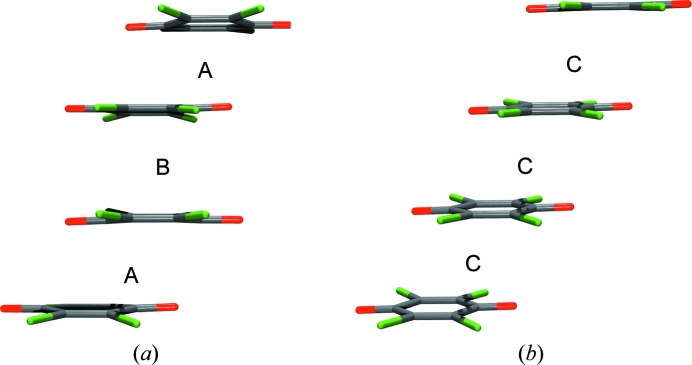
The two types of stacks of semi­quinone radicals. (*a*) A Peierls-distorted stack of radical dimers (alternating longer and shorter interplanar distances). (*b*) A stack of equidistant radicals. Interplanar separations are indicated as A (short, <3.2 Å), B (long, >3.5 Å) and C (intermediate, <3.3 Å). The radicals displayed are tetra­chloro­semi­quinone anions, and the stacks were observed in two polymorphs of the salt with the *N*-methyl­pyridinium cation (Molčanov *et al.*, 2016 ▸).

Planar organic radicals have a great propensity for π stacking. Stacks of different classes of stable radicals (neutral, cations and anions) have been observed: tetra­thia­fulvalene and its derivatives (Rosokha & Kochi, 2007 ▸; Mercuri *et al.*, 2010 ▸; Morita *et al.*, 2013 ▸; Murata *et al.*, 2013 ▸), verdazyls (Rosokha *et al.*, 2010 ▸), phenalenyls (Pal *et al.*, 2004 ▸; Huang & Kertesz, 2007 ▸; Mou *et al.*, 2014 ▸), di­thia­zoles (Beer *et al.*, 2002 ▸), bis­dithia­zolyls (Leitch *et al.*, 2007 ▸), tetra­cyano­pyrazine (Rosokha *et al.*, 2009*a* ▸) *etc*. Two types of stack are known: (i) Peierls-distorted stacks with alternating short (<3.2 Å) and long (>3.4 Å) interplanar separations [Fig. 5 ▸(*a*)] and (ii) stacks of equidistant radicals [typically with interplanar separations <3.3 Å; Fig. 5 ▸(*b*)]. Type (i) is more thermodynamically stable, and it is more common. It can be regarded as stacked radical dimers with coupled spins and such structures are diamagnetic and insulating. The rings in a dimer are bent slightly towards each other; in stacked semi­quinones their Cremer–Pople puckering parameter τ is in the range 2.0–4.3° (Molčanov *et al.*, 2011*a* ▸, 2014*a* ▸, 2019 ▸; Molčanov & Kojić-Prodić, 2017 ▸). Less common is type (ii) with long-range magnetic ordering (usually antiferromagnetic). In this type of stack, the rings are planar within experimental error (Molčanov *et al.*, 2016 ▸). Due to the short distances between the rings, the energy barrier for electron jumping is relatively low, and the crystals are often semiconductors (Itkis *et al.*, 2002 ▸; Lekin *et al.*, 2010 ▸; Mercuri *et al.*, 2010 ▸; Podzorov, 2010 ▸; Yu *et al.*, 2011 ▸, 2012 ▸; Morita *et al.*, 2013 ▸; Murata *et al.*, 2013 ▸; Nakano, 2014 ▸; Chen *et al.*, 2016 ▸). Therefore, they are very interesting for materials chemistry (Lekin *et al.*, 2010 ▸; Mercuri *et al.*, 2010 ▸; Podzorov, 2010 ▸; Sanvito, 2011 ▸; Yu *et al.*, 2011 ▸, 2012 ▸).

**Figure 6:**
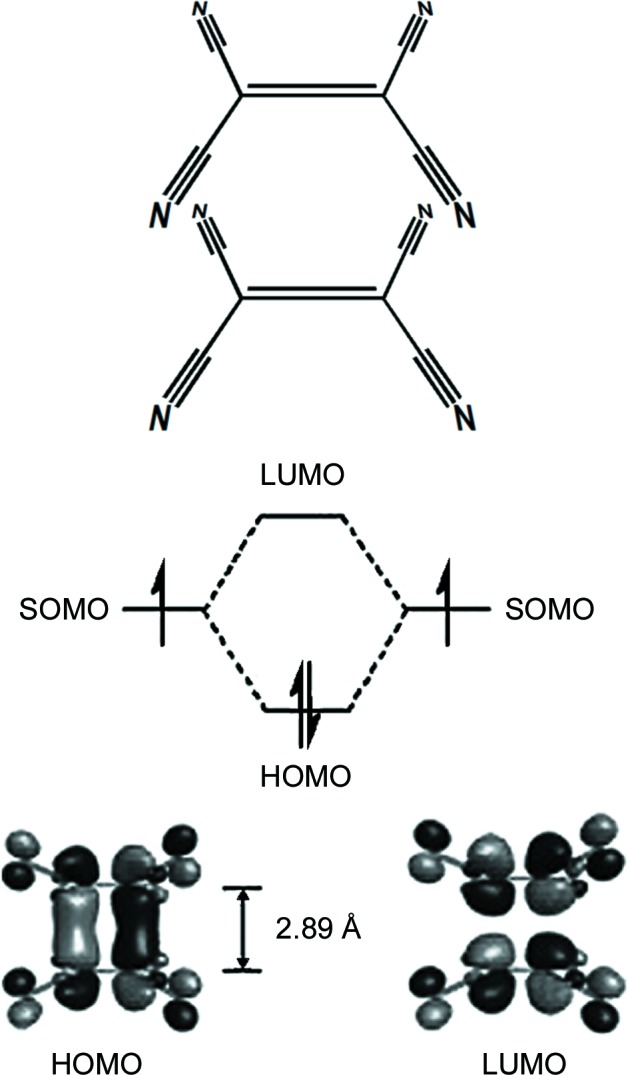
Calculated HOMO and lowest unoccupied molecular orbital (LUMO) in a pair of closely interacting TCNE radical anions, indicating two-electron/multicentric covalent bonding. SOMO denotes a singly occupied molecular orbital. Adapted from Cui *et al.* (2014 ▸ *a*).

It is obvious that the interactions between planar radicals are much stronger than those between closed-shell rings. Since radicals possess unpaired electron(s), it is clear that magnetic exchange and spin coupling make significant contributions to the total interaction. However, the nature and energy of these π interactions remained obscure until quite recently. Quantum chemical studies are available for close dimers of radicals (‘biradicals’) and they indicate a considerable covalent character (Novoa *et al.*, 2009 ▸; Huang *et al.*, 2008 ▸; Cui *et al.*, 2014*a* ▸,*b* ▸; Mou & Kertesz, 2017 ▸; Kertesz, 2018 ▸), with energies ranging from −8 to nearly −20 kcal mol^−1^, but experimental data are lacking. These strong interactions are probably of the same nature as those in dimers of tetra­cyano­ethyl­ene radical anions (TCNE, Fig. 6 ▸) (Novoa & Miller, 2007 ▸; Tian & Kertesz, 2011 ▸; Casado *et al.*, 2013 ▸; Cui *et al.*, 2014 ▸ *a*), which have been interpreted as multicentric covalent bonding. The term ‘pancake bonding’ (Preuss, 2014 ▸; Kertesz, 2018 ▸) has recently been introduced to describe interactions in close dimers of radicals.

Like other planar radicals, semiquinones also form π stacks (Rosokha *et al.*, 2009*b* ▸; Molčanov *et al.*, 2011*a* ▸, 2012 ▸, 2014*a* ▸, 2018*b* ▸; Molčanov & Kojić-Prodić, 2017 ▸) and are prone to Peierls distortion, *i.e.* they readily form pancake-bonded dimers. In our previous work we prepared both types of stack, Peierls-distorted [Fig. 5 ▸(*a*)] and with equidistant radicals [Fig. 5 ▸(*b*)] (Molčanov *et al.*, 2012 ▸, 2016 ▸). Recently, we studied stacks of semi­quinone radicals by X-ray charge-density analysis coupled with DFT calculations (Molčanov *et al.*, 2018*a* ▸, 2019 ▸) on a model system [two polymorphs of a salt of the tetra­chloro­semi­quinone radical anion and the *N*-methyl­pyridinium cation (*N*-MePy·Cl_4_Q)] and provided experimental evidence of pancake bonding (there is a good match between the experimental and calculated electron densities).

#### 2.2.1. Pancake bonding in a dimer of closely interacting radicals (‘biradicals’)   

**Figure 7:**
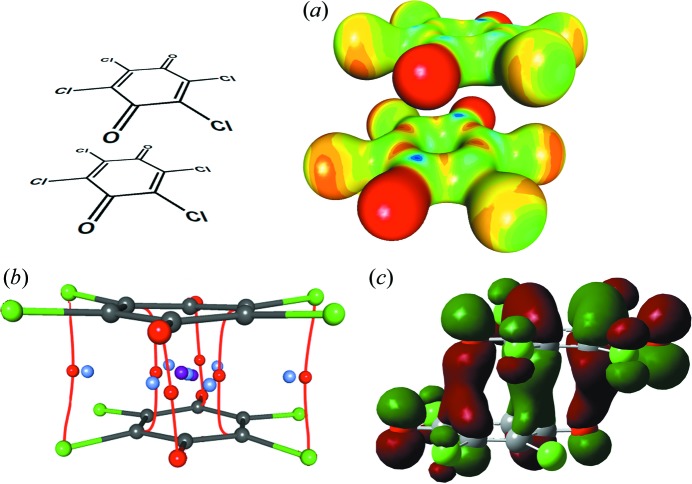
The charge density in a dimer of tetra­chloro­semiquionone radical anions from the salt with the *N*-methyl­pyridinium cation (triclinic polymorph; Molčanov *et al.*, 2019 ▸). (*a*) The experimentally determined electrostatic potential mapped onto an electron-density isosurface of 0.5 e Å^−3^ (red: −0.1, blue: 1.0 e Å^−1^). (*b*) The topology of the experimental electron density. Bonding critical points (3,−1) are depicted as red dots, ring critical points (3,+1) as light blue and cage critical points (3,+3) as violet. (*c*) The HOMO in a dimer calculated by DFT (B3LYP and M06-2X functionals with the def2-QZVPP basis set).

Typical pancake-bonded dimers were found in triclinic *N*-MePy·Cl_4_Q (Molčanov *et al.*, 2016 ▸): the semi­quinone (anion) radicals stack with strictly parallel ring planes, with an interplanar distance of 2.8642 (4) Å and an offset along the O=C⋯C=O axis of 2.072 Å. The electron density between the rings in the dimer reaches almost 0.1 e Å^−3^ (Molčanov *et al.*, 2019 ▸). This is not a high value (for comparison, the maximum electron density in medium-strong hydrogen bonds is about 0.2 e Å^−3^; Molčanov *et al.*, 2015 ▸, 2017*a* ▸) but it extends over a large contact area. Therefore, multiple bonding (3,−1) critical points are found between the rings [Fig. 7 ▸(*b*)] and a cage (3,+3) critical point (a local minimum of electron density) is found in the centre. The integrated electron density obtained by DFT calculation exceeds 1 e, and the corresponding calculated bond order is 0.80 (Molčanov *et al.*, 2019 ▸). The highest occupied molecular orbital (HOMO) extends between the two rings [Fig. 7 ▸(*c*)].

The energy of the interaction was evaluated by DFT calculations (B3LYP and M06-2X functionals with the def2-QZVPP basis set) and the total is repulsive; however, it is compensated by the cations (in a tetramer comprising two cations and two anions, the total energy is strongly attractive). In the crystal structure, local repulsions are overcome by Madelung energy. It is interesting to note that the covalent contribution (SOMO–SOMO interaction, where SOMO denotes a singly occupied molecular orbital) is −9.4 kcal mol^−1^; this value is similar to other pancake bonds studied by computational methods (Novoa *et al.*, 2009 ▸; Tian & Kertesz, 2011 ▸; Cui *et al.*, 2014*a* ▸,*b* ▸; Mou & Kertesz, 2017 ▸; Kertesz, 2018 ▸). Due to the partially covalent nature of intradimer interactions, the spins are paired and the ground state is singlet. Therefore, a pancake bond can be regarded as an unlocalized two-electron/multicentric covalent bond.

It is also evident that electrostatic interactions play an integral part in pancake bonding: a map of the electrostatic potential shows that the closest contacts are between the most electron-rich (carbonyl oxygens) and the most electron-depleted (carbonyl carbons) parts of contiguous rings [Fig. 7 ▸(*a*)]. Such an arrangement minimizes the electrostatic repulsion between two negative charges.

In the longer contact between the dimers [interplanar separation 3.5993 (4) Å], there is very low electron density (<0.04 e Å^−3^) and the calculated intramolecular bond order is a negligible 0.04 (Molčanov *et al.*, 2019 ▸). Therefore, the inter-dimer interaction comprises mostly dispersion interactions and is similar to the weak π interaction between aromatic rings.

#### 2.2.2. Pancake bonding in a trimer of closely interacting partially charged radicals   

While pancake-bonding radical dimers are well known, a handful of examples of pancake-bonded trimers have also been reported (Ashwell *et al.*, 1977 ▸; Endres *et al.*, 1978 ▸; Nishijo *et al.*, 2004 ▸; Akutagawa *et al.*, 2004 ▸; Shvachko *et al.*, 2016 ▸; Zhang *et al.*, 2016 ▸). These trimers possess two unpaired electrons which are shared between three radicals; quantum models have proposed partially covalent interactions extending between three rings (Takamuku *et al.*, 2017 ▸). They are similar to, but somewhat weaker than, pancake bonding in a dimer; the ground state is also singlet.

**Figure 8:**
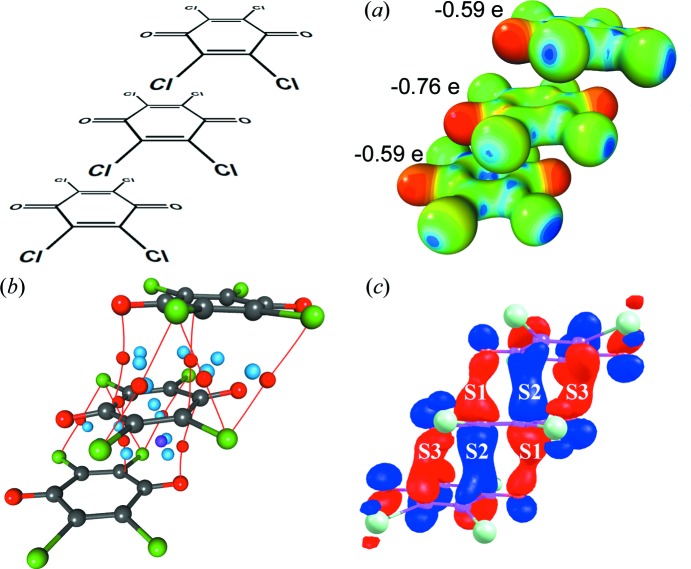
The charge density in a trimer of tetra­chloro­semiquionone radical anions from the salt with the 4-di­methyl­amino-*N*-methyl­pyridinium (4-damp) cation (Molčanov *et al.*, 2018*a* ▸). (*a*) The experimentally determined electrostatic potential mapped onto an electron-density isosurface of 0.5 e Å^−3^ (red: −0.1, blue: 1.0 e Å^−1^). (*b*) The topology of the experimental electron density between two rings in a trimer. Bonding critical points (3,−1) are depicted as red dots, ring critical points (3,+1) as light blue and cage critical points (3,+3) as violet. (*c*) The HOMOs in a dimer calculated by DFT [M05-2X/6-311G(d,p)].

X-ray charge-density analysis of a trimer of tetra­chloro­semi­quinones (Molčanov *et al.*, 2018*a* ▸; Fig. 8 ▸) provided experimental evidence for this concept. The total charge of the trimer is −1.94 (very close to the formal value of −2), but the charge is distributed unevenly: the central ring has a charge of −0.76, while the two lateral ones each have a charge of −0.59. The semi­quinones are offset along the O=C⋯C=O axis (‘longitudinal offset’; Rosokha *et al.*, 2009*b* ▸; Molčanov & Kojić-Prodić, 2017 ▸) to minimize electrostatic repulsion [Fig. 8 ▸(*a*)], and numerous bonding (3,−1) critical points are found between the rings [Fig. 8 ▸(*b*)], with a maximum electron density of 0.77 e Å^−3^. Also, a (3,+3) critical point (a local minimum of electron density) is found in each of the symmetry-equivalent close contacts between the rings in the trimer [Fig. 8 ▸(*c*)], indicating a cage-like electronic structure.

Molecular orbital calculations by DFT [M05-2X/6-311G(d,p)] indicated that the HOMO extends between all three rings of the trimer; the covalent contribution to the total interaction between each pair of rings was calculated by DFT [M05-2X/6-311G(d,p)] to be −6.8 kcal mol^−1^ (Molčanov *et al.*, 2018*a* ▸), which is somewhat less than in a pancake-bonded dimer (see above), and the bond order was estimated to be lower than 0.71 (most likely it is about 0.5).

A long intertrimer contact reveals a low electron density (*ca* 0.04 e Å^−3^) and its geometry is similar to the stacking of aromatic rings.

#### 2.2.3. Partially covalent nature of interactions in stacks of equidistant radicals   

In stacks of equidistant radicals the interplanar distance is mostly <3.3 Å, which is longer than for the pancake bonds in dimers, but still much shorter than the long contacts between dimers. Antiferromagnetism and semiconductivity indicate some kind of long-range interaction, but studies of this type of stacking are few (Molčanov *et al.*, 2016 ▸).

**Figure 9:**
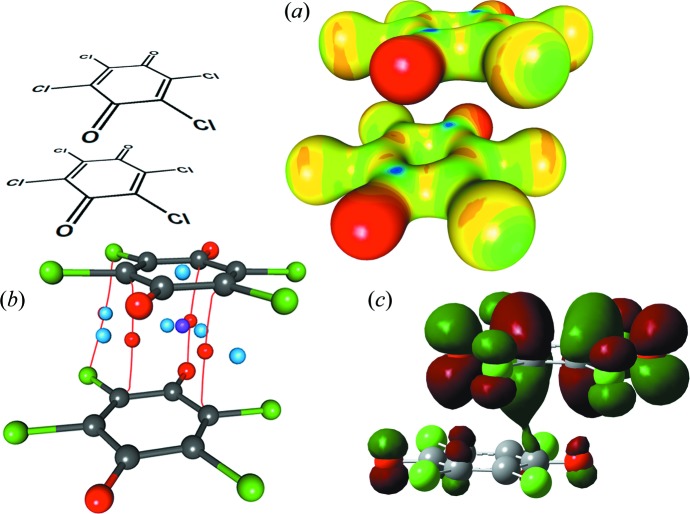
The charge density in a stack of equidistant tetra­chloro­semiquionone radical anions from the salt with the *N*-methyl­pyridinium cation (orthorhombic polymorph; a pair of contiguous radicals is shown; Molčanov *et al.*, 2019 ▸). (*a*) The experimentally determined electrostatic potential mapped onto an electron-density isosurface of 0.5 e Å^−3^ (red: −0.1, blue: 1.0 e Å^−1^). (*b*) The topology of the experimental electron density. Bonding critical points (3,−1) are depicted as red dots, ring critical points (3,+1) as light blue and cage critical points (3,+3) as violet. (*c*) The HOMOs calculated by DFT (B3LYP and M06-2X functionals with the def2-QZVPP basis set).

Our X-ray charge-density study of the orthorhombic polymorph of the *N*-methyl­pyridinium salt of tetra­chloro­semi­quinone (Molčanov *et al.*, 2019 ▸) [equidistant stacks with interplanar separations of 3.1688 (6) Å] revealed relatively little electron density between the rings (maximum 0.048 e Å^−3^) and fewer bonding (3,−1) critical points than in the pancake-bonded dimer, but the (3,+3) local minimum was present [Fig. 9 ▸(*b*)]. This indicates a significantly weaker covalent contribution than in the pancake-bonded dimers, but nevertheless considerably stronger interaction than between the dimers or between aromatic rings. However, this electron density is consistent with DFT calculations (B3LYP and M06-2X functionals with the def2-QZVPP basis set), which indicated a weak covalent contribution [Fig. 9 ▸(*c*)] with a bond order of 0.26. While the calculation was performed for a pair of radicals only, we can be quite certain that the HOMOs extend along the stack, resulting in some kind of metal-like state. Therefore, we can consider the interactions in a stack of equidistant radicals as weak pancake bonding and conclude that the covalent contribution is critical for semiconductivity.

The offset along the O=C⋯C=O axis of 2.057 Å [Fig. 9 ▸(*a*)] is nearly identical to that in pancake-bonded dimers, and minimizes electrostatic repulsion.

### 2.3. Stacking of metal-chelate rings: electrostatic interactions   

**Figure 10:**
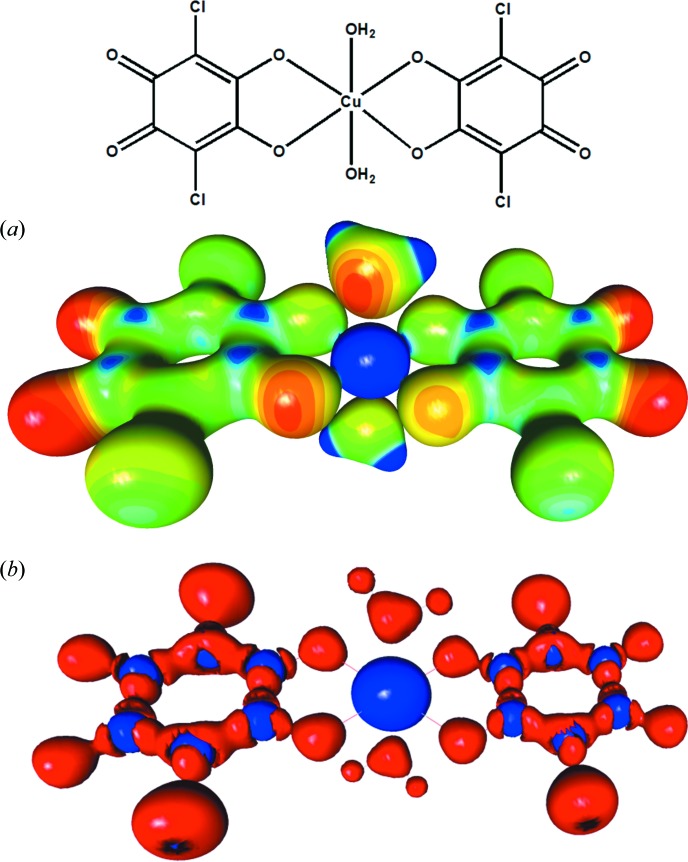
The charge density in the [Cu(CA)_2_(H_2_O)_2_]^2+^ cation from [Cu(CA)_2_(H_2_O)_2_]im_2_ (Jurić *et al.*, 2016 ▸; Vuković *et al.*, 2019 ▸). (*a*) The experimentally determined electrostatic potential mapped onto an electron-density isosurface of 0.5 e Å^−3^ (red: −0.1, blue: 1.0 e Å^−1^). (*b*) The Laplacian of the electron density (red denotes negative and blue positive).

It is known that metal-chelate rings sometimes participate in π stacking, often interacting with aromatic rings, and they are quite often mentioned in the literature (Kravtsov, 2004 ▸; Molčanov *et al.*, 2007 ▸, 2013*a* ▸, 2014*b* ▸; Babić *et al.*, 2008 ▸; Androš *et al.*, 2010 ▸; Jurić *et al.*, 2016 ▸; Malenov *et al.*, 2017 ▸). Some authors have even claimed that the observed π stacking was evidence that their metal-chelate rings were aromatic (Castiñeiras *et al.*, 2002 ▸; Karabıyık *et al.*, 2010 ▸). While metallo­aromaticity is a somewhat contentious issue (Milčić *et al.*, 2007 ▸; Feixas *et al.*, 2013 ▸; Masui, 2014 ▸; Malenov *et al.*, 2017 ▸), it may be expected only in some rare cases of complexes of transition metals with unsaturated α,α′- and β,β′-ligands (Masui, 2014 ▸); there is no way that chelate rings with decidedly non-aromatic ligands, such as 2,5-DHQs, can display metallo­aromaticity. Moreover, accurate X-ray charge-density data confirmed the nature of metal–ligand bonds for first-row transition metals and the most common *N*- and *O*-donor ligands: due to a low electron density at the critical points (mostly >0.5 e Å^−3^) and positive values of the Laplacian, they can be classified as borderline cases between ionic and highly polar covalent interactions (Poulsen *et al.*, 2004 ▸; Pillet *et al.*, 2004 ▸; Bianchi *et al.*, 2005 ▸; Wang, 2014 ▸; Vologzhanina *et al.*, 2015 ▸; Chuang *et al.*, 2017 ▸; Gajda & Woźniak, 2017 ▸). *M*—O bonds in Cu and Mn complexes of 2,5-DHQs [Fig. 10 ▸(*b*)] are no exception (Vuković *et al.*, 2019 ▸), so these chelate rings are definitely not aromatic. Since we have proven that aromaticity is not a *conditio sine qua non* for stacking, but that non-aromatic rings also stack (Molčanov *et al.*, 2009*b* ▸, 2011*a* ▸, 2013*a* ▸, 2014*b* ▸, 2015 ▸; Jurić *et al.*, 2016 ▸), we can dismiss claims that the metal-chelate rings are aromatic because they stack.

Our work has revealed various types of π stacking between all kinds of rings in complexes of transition metals with 2,5-DHQs and aromatic *N*-donor ligands {for example, [Cu(CA)(2,2′-bpy)] (Molčanov *et al.*, 2013*a* ▸) and [Cu(CA)(MeCN)]*_n_* (Jurić *et al.*, 2016 ▸)}: aromatic⋯quinoid, aromatic⋯metal chelate, metal chelate⋯metal chelate, quinoid⋯metal chelate *etc.* (Molčanov *et al.*, 2013*a* ▸, 2014*b* ▸; Jurić *et al.*, 2016 ▸). Their geometry is similar to common aromatic stacking, but the interplanar distances are often shorter than 3.4 Å (Molčanov *et al.*, 2013*a* ▸, 2014*b* ▸; Jurić *et al.*, 2016 ▸).

**Figure 11:**
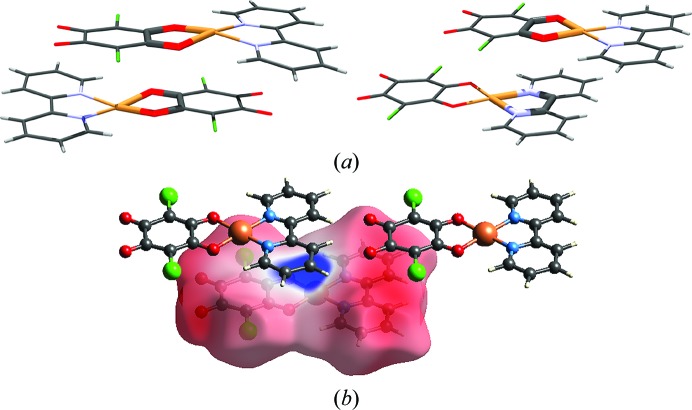
The stacking in [Cu(CA)(2,2′-bpy)] (Molčanov *et al.*, 2013*a* ▸). (*a*) Pairs of contiguous molecules with highlighted close contacts between metal-chelate rings. (*b*) The electrostatic potential plotted onto a Hirshfeld surface. It is apparent that there is close contact between the electron-rich (red) and electron-poor (blue) regions.

The accurate high-resolution X-ray diffraction data that would be required for charge-density studies of compounds with stacked metal-chelate rings are still lacking, but some insight into their π interactions can be gained by analysis of the Hirshfeld surface (HS) (Hirshfeld, 1977 ▸; Spackman *et al.*, 2008 ▸; Spackman & Jayatilaka, 2009 ▸) and Voronoi–Dirichlet polyhedra (VDP) (Blatov, 2004 ▸). In the crystal packing of the planar complex [Cu(CA)(2,2′-bpy)] (bpy = 2,2′-bi­pyridine; CA = chloranilate), metal chelate⋯metal chelate and quinoid⋯metal chelate contacts have been observed (Molčanov *et al.*, 2013*a* ▸) [Fig. 11 ▸(*a*)]. The molecular planes are close to parallel (α = 2°), with an interplanar distance of 3.28 Å and centroid-to-centroid distances ranging between 3.40 and 3.84 Å. The electrostatic potential plotted onto an HS reveals excess negative charge at the bpy and chloranilate entities [Fig. 11 ▸(*b*)], while the central Cu atom has a considerable positive charge. This is in a good agreement with the electrostatic potential of the Cu–CA complex [Cu(CA)_2_(H_2_O)_2_]im_2_ (im = imidazolium) derived from high-resolution X-ray data [Fig. 10 ▸(*a*)] (Vuković *et al.*, 2019 ▸). The molecules in the crystal structure are arranged to form close contacts between the electron-depleted (Cu-chelate) and electron-rich (bpy and chloranilate) parts of contiguous molecules. Therefore, it is apparent that the main component of the interaction between the rings is electrostatic. However, since the total area of an intermolecular contact is rather large, the total interactions must be quite strong, consistent with short interplanar separations.

**Figure 12:**
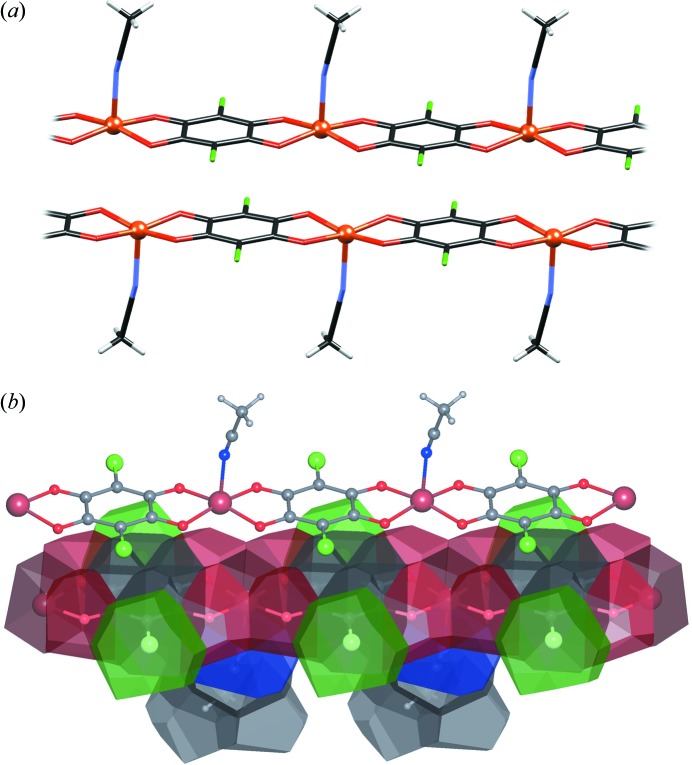
The stacking in [Cu(CA)(MeCN)]*_n_* (Jurić *et al.*, 2016 ▸). (*a*) A pair of contiguous molecules. (*b*) The molecular surface represented as VDP.

Similar interactions exist between 1D coordination polymers [Cu(CA)(MeCN)]*_n_* (Jurić *et al.*, 2016 ▸) [Fig. 12 ▸(*a*)]: the polymer chains run parallel, with an interplanar separation of 3.26 Å and an offset of *ca* 1.6 Å normal to the direction of the polymer. Again, the closest contacts are between electron-depleted Cu and electron-rich chloranilate rings [Fig. 12 ▸(*b*)], and the contact surfaces of two contiguous chains are relatively flat, comprising a multitude of small facets [Fig. 12 ▸(*c*)].

**Figure 13:**
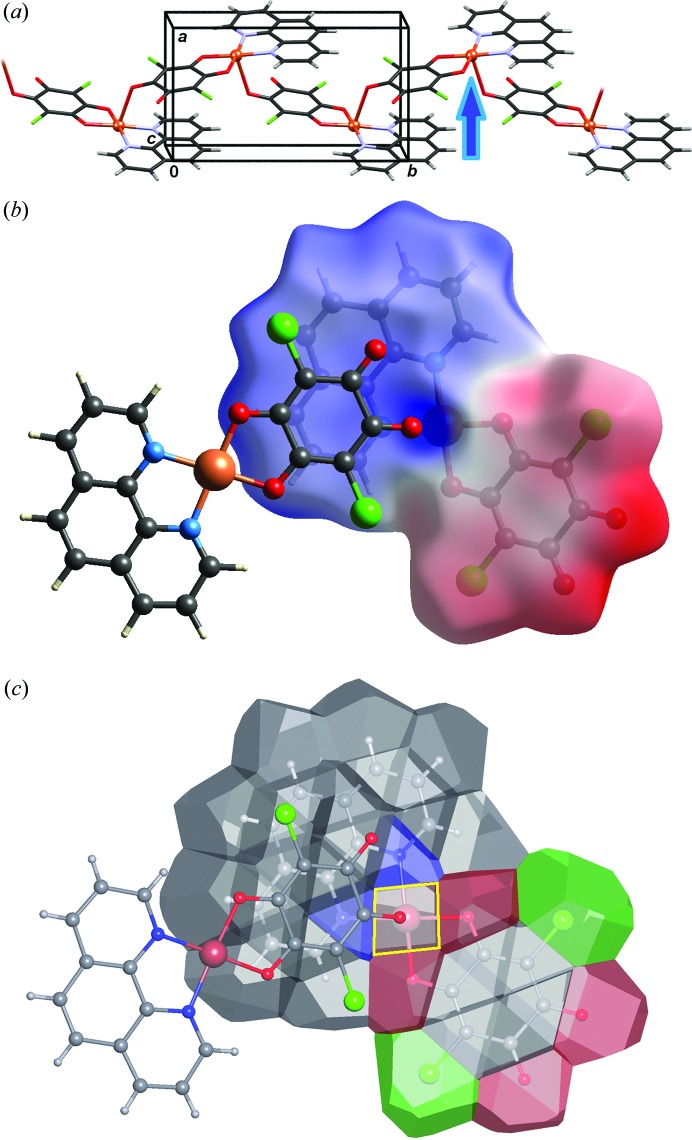
The stacking in [Cu(CA)(phen)]*_n_* (Molčanov *et al.*, 2014*b* ▸). (*a*) A polymeric chain with a long Cu—O bond indicated by a blue arrow. (*b*) The electrostatic potential plotted onto a Hirshfeld surface. It is apparent that there is close contact between the electron-rich (red) and electron-poor (blue) regions. (*c*) The molecular surface represented as VDP. The face corresponding to the elongated Cu—O bond is highlighted in yellow.

A curious case of a borderline phenomenon between coordination bonding and π stacking was noted in [Cu(CA)(phen)]*_n_* [phen = 1,10-phenanthroline; Fig. 13 ▸(*a*)]: the Cu⋯O contact of 2.574 (2) Å is much shorter than the sum of the van der Waals radii, but nevertheless significantly longer than the sum of the covalent radii (Molčanov *et al.*, 2014*b* ▸). The splitting of the C—O stretching bands in the IR spectrum (by 6 cm^−1^) indicated weak bonding between Cu and O, leading to the conclusion that the Cu coordination is in fact 4+1, making this one of the longest examples of a Cu—O bond. Analysis of the VDP [Fig. 13 ▸(*b*)] shows that the face corresponding to the long Cu—O bond is *ca* 50% smaller than the faces representing the other four Cu—O bonds, but more than twice as large as typical faces representing intermolecular contacts.

## 3. Conclusions   

**Figure 14:**
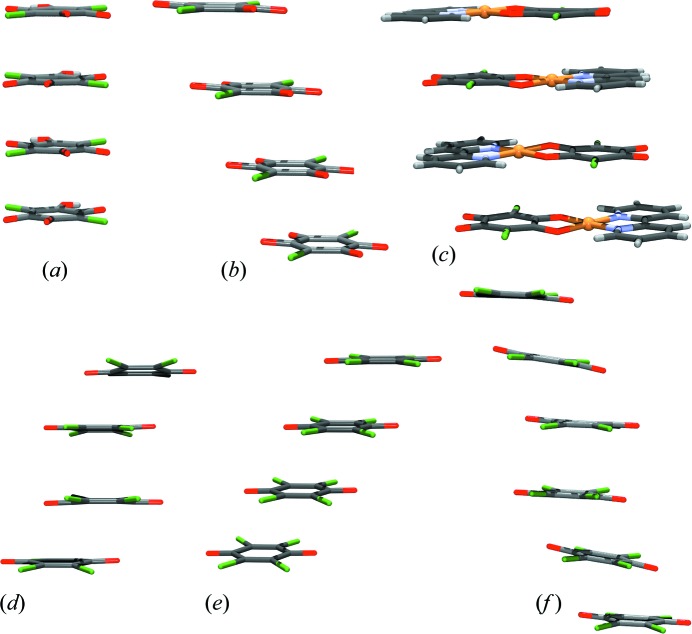
Stacks of non-aromatic rings. (*a*) Hydrogen chloranilate anions in KHCA·2H_2_O (Molčanov *et al.*, 2009*b* ▸), (*b*) chloranilate dianions in Na_2_CA·2H_2_O (Molčanov *et al.*, 2009*a* ▸), (*c*) [Cu(CA)(2,2′-bpy)] (Molčanov *et al.*, 2013*a* ▸), (*d*) pancake-bonded dimers of tetra­chloro­semi­quinone radical anions in triclinic *N*-MePy·Cl_4_Q (Molčanov *et al.*, 2016 ▸), (*e*) equidistant tetra­chloro­semi­quinone radical anions in orthorhombic *N*-MePy·Cl_4_Q (Molčanov *et al.*, 2016 ▸) and (*f*) triplets of partially charged tetra­chloro­semi­quinone radicals in (4-damp)_2_(Cl_4_Q)_3_ (Molčanov *et al.*, 2018*a* ▸). Stacks (*a*), (*b*) and (*c*) comprise closed-shell rings, while (*d*), (*e*) and (*f*) are radicals.

We have presented a stacking model of planar polyenic systems which is primarily based on experimentally determined charge density, previously published by us (Molčanov *et al.*, 2015 ▸, 2018*a* ▸, 2019 ▸; Molčanov & Kojić-Prodić, 2017 ▸). It is applicable to planar rings, regardless of π-electron delocalization. Generally, π stacking covers a wide range of energies and types of interaction, from dispersion to weak unlocalized covalent bonding (Fig. 14 ▸). The energies involved are in the range <1 kcal mol^−1^ (typical for aromatics) to >15 kcal mol^−1^ (‘pancake bonding’ between the radicals).

It is interesting to note that, among closed-shell molecules, the weakest interactions are between fully delocalized π systems (*i.e.* aromatics) and the strongest are between rings with little or no electron delocalization (such as quinones).

One can summarize: (i) in common stacking of aromatic rings, dispersion and electric quadrupoles are dominant; (ii) in stacking of quinones (and other non-aromatic polyenic systems), the prevailing interaction is (multipolar) electrostatic; and (iii) in stacking of metal chelate rings, the prevailing interaction is also electrostatic (multipolar), with the possible contribution of coordination bonding.

Planar stacked radicals reveal three possible types of interaction: (i) covalent ‘pancake bonding’, with a considerable electrostatic component (radical dimers); (ii) electrostatic, with a non-negligible covalent component (stacks of equidistant radicals); and (iii) dispersion (between pancake-bonded dimers). As our analysis of charge densities indicates, there is no clear-cut border between all these types. In addition, we may establish an AIM criterion for recognizing pancake bonding: a local electron-density minimum [a (3,+3) critical point] should exist between two radicals, while the electron density in multiple (3,−1) critical points should exceed 0.7 e Å^−3^.

Stacking interactions exhibit characteristics comparable with those of hydrogen bonding. They both cover a broad range from dispersion (the weakest hydrogen bonds, such as C—H⋯S and C—H⋯Cl) to two-electron/three-centric covalent bonding (the strongest hydrogen bonds, such as in the Zundel cation) (Steiner, 2002 ▸; Molčanov *et al.*, 2017*a* ▸).

Intermolecular interactions involving a charge transfer (proton and/or electron) are accompanied by stacking interactions and/or hydrogen bonding, and both exhibit a certain degree of covalent bonding – multicentric bonding (Molčanov *et al.*, 2018*b* ▸, 2019 ▸). This means that the basic definition of noncovalent interactions should be used more carefully.

The predominant interaction in a particular stacked system modulates the physical properties and defines a strategy for crystal engineering of functional materials. The separation distance of stacked quinoid rings correlates with their magnetic characteristics and thus the occurrence of coupled/uncoupled electron spins (Molčanov *et al.*, 2012 ▸, 2016 ▸, 2018*a* ▸,*b* ▸, 2019 ▸). In an equidistant array of stacked quinoid rings, antiferromagnetic or semiconducting properties can be produced depending on the electron-transfer energy within the stack (Molčanov *et al.*, 2012 ▸, 2016 ▸, 2018*b* ▸). However, having a plethora of interactions within a stack, as also revealed by examples discussed in this work, requires fine tuning of the crystal-engineering procedures to prepare structures with selective and sensitive properties.
